# Description of trauma among French service members in the Department of Defense Trauma Registry: understanding the nature of trauma and the care provided

**DOI:** 10.1186/s40779-019-0197-6

**Published:** 2019-02-27

**Authors:** Marc A. Schweizer, Jud C. Janak, Zsolt T. Stockinger, Tristan Monchal

**Affiliations:** 1 United States Department of Defense Joint Trauma System, Joint Base San Antonio Fort Sam Houston, Houston, TX 78234 USA; 2 Naval Medical Readiness Training Command Jacksonville, Jacksonville, FL 32212 USA; 3 Sainte Anne Military Hospital, 600-83800 Toulon Cedex 9, BP France

**Keywords:** French service members, U.S. military treatment facility, Trauma registry

## Abstract

**Background:**

Since 2001, the French Armed Forces have sustained many casualties during the Global War on Terror; however, even today, there is no French Military trauma registry. Some French service members (SMs) were treated in US Military Medical Treatment Facilities (MTFs) and were recorded in the US Department of Defense Trauma Registry (DoDTR). Our objective was to conduct a descriptive analysis of the injuries sustained by French SMs reported in the DoDTR and subsequent care provided to them to assist in understanding the importance of building a French Military trauma registry.

**Methods:**

Using DoDTR data collected from 2001 to 2017, a retrospective descriptive analysis was conducted. We identified 59 French SMs treated in US MTFs. The characteristics of the SMs’ demographics, injuries, care provided to them, and discharge outcomes were summarized.

**Results:**

Among the 59 French SMs identified, 46 (78%) sustained battle injuries (BIs) and 13 (22%) sustained nonbattle injuries (NBIs). There were 47 (80%) SMs injured in Afghanistan (*Opération Pamir)*, while 12 (20%) were injured in *Opération Chammal* in Iraq and Syria. Explosives accounted for 52.5% of injuries, while 25.4% were due to gunshot wounds; all were BIs. The majority of reported injuries were penetrating (59.3%), most of which were BIs (71.7%). The mean Injury Severity Score for BIs was 12 (SD = 8.9) compared to 6 (SD = 1.7) for NBIs. Around half of SMs (*n =* 30; 51%) were injured in Afghanistan between the years 2008–2010. Among a total of 246 injuries sustained by 59 patients, extremities were the body part most prone to BIs followed by the head and face. Four SMs died after admission (6.8%).

**Conclusions:**

The DoDTR provides extensive data on trauma injuries that can be used to inform injury prevention and clinical care. The majority of injuries sustained by French SMs were BIs, caused by explosives, and predominantly occurring to the extremities; these findings are similar to those of other studies conducted in combat zones. There is a need to establish a French Military trauma registry to improve the combat casualty care provided to French SMs, and its creation may benefit from the DoDTR model.

## Introduction

Since the start of the Global War on Terror in 2001, French military forces have been part of the international military coalitions’ forces in different operations in Afghanistan and the Middle East (i.e. Iraq and Syria). From 2001 to 2014, France was involved in *Opération Pamir* as a part of the *International Security and Assistance Force (ISAF)* in Afghanistan led by the *North Atlantic Treaty Organization (NATO)*, which ran concurrently with its US counterpart, *Operation Enduring Freedom (OEF)* that began in October 2001 [1, 2]. Within the ISAF, the French forces participated under *Opération Héraclès* and *Opération Arès*. French forces were also involved in *Opération Epidote* that aimed to provide training and support to the Afghan Army. France assumed command of the medical hospital at the Kabul International Airport (KaIA) located in the capital region of Afghanistan from 2009 to 2014 [3]. This NATO hospital provided Role 3 medical and surgical capability care to Coalition and Afghan patients [4]. From 2014 onward, France became involved in the international coalition against terrorism in Iraq and Syria in *Opération Chammal* alongside the American counterpart *Operation Inherent Resolve (OIR).*

The conflicts associated with the Global War on Terror were and continue to be responsible for a significant number of combat-related injuries among coalition military forces, including French service members (SMs) [5]. The French Military Center for Epidemiology and Public Health *(Centre d’Epidémiologie et de Santé Publique des Armées)* collects all ballistic and explosive injury data from weekly healthcare reports provided by deployed French military treatment facilities (MTFs). However, these reports tend to be selective, heterogeneous, and reporter-dependent [6]. Since 2003, the French Military Health Service *(Service de Santé des Armées)* [7] has managed an electronic database that contains a prospective surgical record of procedures performed by deployed surgeons at Role 2 (Forward Surgical Teams that provide primary care and basic emergency treatment) and Role 3 (Combat Support Hospitals that provide the highest level of medical care in combat zones) levels. This registry was the source of several studies that focused on the surgical workload of French MTFs (e.g., Bonnet et al., 2012 [5]; Barbier et al., 2014 [3]; Malgras et al., 2016 [8]; de Lesquen et al., 2016 [9]; Beranger et al., 2017 [10]; Barbier et al., 2017 [11]).

Since 2001, the United States Department of Defense Trauma Registry (DoDTR), formerly the *Joint Theater Trauma Registry (JTTR)*, has collected data on traumatic injuries sustained by any patient treated in US MTFs as well as information regarding patient demographics and care provided [12, 13]. Those treated in these MTFs can be US SMs, their beneficiaries, non-US coalition SMs, host nation security forces, and local civilians. The DoDTR is the largest military medical trauma database in existence. It provides crucial evidence necessary to support clinical research and performance improvements throughout military medicine and contributes to medical advances in combat casualty care. To our knowledge, there are no studies regarding French SMs treated in US MTFs, and there is a knowledge gap in regard to their experiences before being discharged or transferred to French MTFs. The French Military may potentially benefit from a descriptive summary of injury pattern of these casualties. Moreover, such a summary would illustrate the importance of the multinational nature of medical support that is now part of modern operations. The objective of this study was to describe the injuries sustained by French SMs treated in US MTFs and subsequent care provided to them in order to understand the importance of creating a French Military trauma registry based on the JTS DoDTR model.

## Methods

Using data from the DoDTR that was collected from 2001 to 2017, we conducted a retrospective descriptive analysis. The DoDTR is a US military trauma registry that is maintained by the Joint Trauma System (JTS) [14] and contains data from abstracted medical records of patients who sustained traumatic injuries and were treated in US MTFs. There were two primary inclusion criteria for this analysis, as follows: (1) French SMs who sustained traumatic injuries and (2) treatment must have occurred in at least one US MTF, regardless of location, cause of injury, or the operation in which they were involved. We intended to examine the traumatic injuries in deployed settings; therefore, French civilians were excluded from this study. The patients who were included were stratified into two groups based on whether the injuries sustained had occurred in battle or nonbattle settings.

For each group (i.e., battle vs. nonbattle), we conducted a descriptive summary of patient demographics, injury characteristics (e.g., injured body region, injury severity, etc.), and medical interventions (e.g., type of procedures done, type and amount of resuscitative fluids and blood products given). We plotted the injuries per year in order to provide a visualization of injury trends. We used the Abbreviated Injury Scale (AIS) to identify injured body regions and to calculate the overall Injury Severity Score (ISS) [15]. When describing categorical variables, we reported the total number and percent stratified by battle status, while for continuous variables, the mean and the standard deviation (SD) were reported. Chi-square tests were used for categorical variables, while Student’s *t*-tests were used for continuous variables. A *p*-value of 0.05 was used to define statistical significance*.* The injuries sustained per body region were plotted against their severity to provide a better understanding of which body region sustained more severe injuries in this particular population. We also looked into some of these characteristics per MTF Role in order to examine the characteristics of the injuries and the care provided by the MTFs’ perspective. We defined prehospital transportation time as the time difference in minutes between reported injury time and arrival time to the first US MTF, as reported in the DoDTR. We used SAS® version 9.4 (SAS® Institute Inc., Cary, NC) to conduct our analyses. This quality improvement project was deemed exempt from institutional board review by the US Army Institute of Surgical Research because it was not designed to contribute to generalizable knowledge, and therefore, it did not constitute research as defined in 32 CFR 219.102(d).

## Results

There were 59 French SMs who sustained traumatic injuries and were treated in US MTFs, of these, 46 (78%) were battle injuries (BIs) and 13 (22%) were nonbattle injuries (NBIs) (Table 1). The mean age was 30.6 years (SD = 7.6); those with BIs were on average 29.1 years old (SD = 6.9) compared to 35.5 years (SD = 8) for those with NBIs. All of the 59 injured SMs were male. There were 47 (80%) French SMs injured in *Opération Pamir* (reported in the DoDTR as Operation Enduring Freedom), while 12 (20%) were injured in *Opération Chammal* (reported as Operation Inherent Resolve). More than half of all injuries were caused by explosives (52.5%), while a quarter (25.4%) were due to gunshots; all of these causes were battle-related. NBIs consisted of crushing, falls, fires or flames, helicopter crashes, motor vehicle crashes, and sharp objects.

**Table 1:** Demographic characteristics of French service members treated in US Military Medical Treatment Facilities

Characteristics	Battle *n =* 46 (78%)	Non-Battle *n =* 13 (22%)	Total *n =* 59	*p*-value
Age^a^				0.08
19-24	12 (26.1%)	–	12 (20.3%)	
25–29	12 (26.1%)	5 (38.4%)	17 (28.8%)	
30–34	11 (23.9%)	2 (15.4%)	13 (22%)	
35–39	3 (6.5%)	2 (15.4%)	5 (8.5%)	
40–48	4 (8.7%)	4 (30.8%)	8 (13.6%)	
Unknown	4 (8.7%)	–	4 (6.8%)	
Mean ± SD^b^	29.1 ± 6.9	35.5 ± 8.0	30.6 ± 7.6	
Gender				–
Male	46 (100%)	13 (100%)	59 (100%)	
Female	–	–	–	
Military Operation				0.29
OEF^c^	38 (82.6%)	9 (69.2%)	47 (79.7%)	
OIR^d^	8 (17.4%)	4 (30.8%)	12 (20.3%)	
Mechanism of Injury				< 0.01
Explosive	31 (67.4%)	–	31 (52.5%)	
Gunshot wound	15 (32.6%)	–	15 (25.4%)	
Crush	–	1 (7.7%)	1 (1.7%)	
Fall	–	2 (15.4%)	2 (3.4%)	
Fire/Flame	–	1 (7.7%)	1 (1.7%)	
Helicopter crash	–	2 (15.4%)	2 (3.4%)	
Knife/Sharp object	–	2 (15.4%)	2 (3.4%)	
Motor Vehicle Crash	–	5 (38.4%)	5 (8.5%)	
Predominant Injury Type				< 0.01
Penetrating	33 (71.7%)	2 (15.4%)	35 (59.3%)	
Blunt	7 (15.2%)	10 (76.9%)	17 (28.8%)	
Burn	6 (13.1%)	1 (7.7%)	7 (11.9%)	
Injury Severity Score				0.29
1–8	20 (43.5%)	9 (69.2%)	29 (49.1%)	
9–15	12 (26.1%)	3 (23.1%)	15 (25.4%)	
16–25	8 (17.4%)	1 (7.7%)	9 (15.3%)	
> 25	6 (13%)	–	6 (10.2%)	
Mean ± SD^b^	12 ± 8.9	5.8 ± 1.7	10.6 ± 8.0	

^a^ .Age Measured in Years; ^b^ .Standard Deviation; ^c^. Operation Pamir Reported in DoDTR as Operation Enduring Freedom; ^d^ . Operation Chammal Reported in DoDTR as Operation Inherence Resolve

**Fig. 1:**
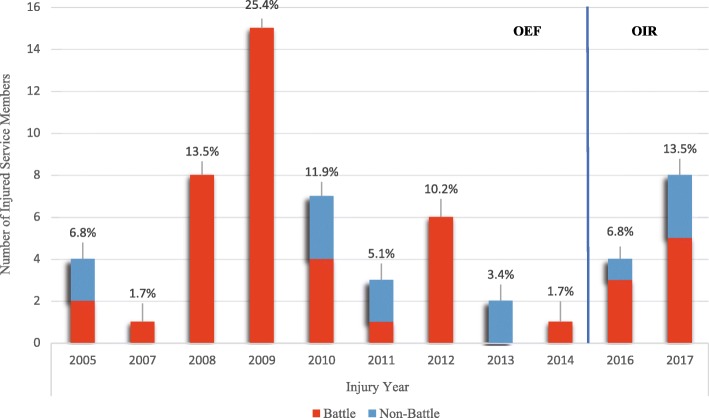
Counts and Percentages of French Service Members Treated in US Military Treatment Facilities (2005–2017). *OER* Operation Enduring Freedom, *OIR* Operation Inherent Resolve

**Fig. 2:**
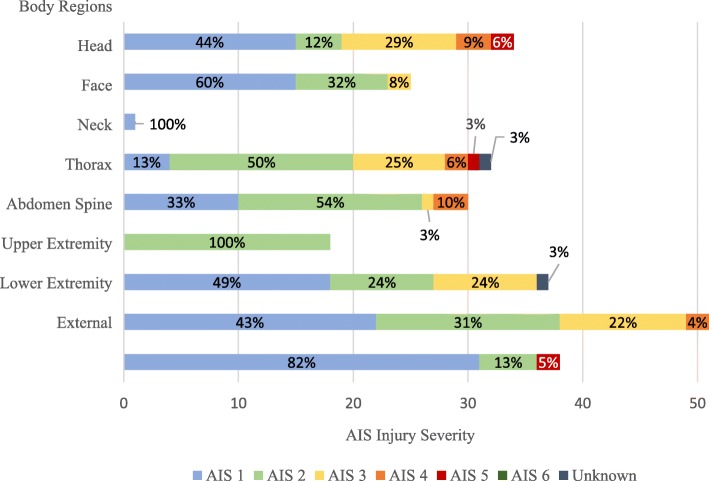
Proportions of Injuries among French Service Members Treated in US Military Treatment Facilities per Body Region and AIS Severity. *AIS* Abbreviated Injury Scale

BIs accounted for 88% of all injuries (217 of 246) with an average of 5 injuries per person, while NBIs (*n =* 29) were approximately 12% of all injuries with an average of 2 injuries per person (Table 2). Around one-quarter of all injuries (25.4%) happened in 2009, while the fewest occurred in 2007 and 2014 (1.7% each). All of the injuries in the aforementioned years were in battle settings (Fig. 1). More than half of all SMs (*n =* 30; 51%) were injured during the years 2008–2010, with only three (5.1%) occurring in nonbattle settings. Most reported injuries were penetrating in nature (59.3%), of which the majority were in battle settings (71.7%; *p*-value: < 0.01). The overall mean Injury Severity Score (ISS) was 10.6 (SD = 8), with around half of all SMs sustaining minor injuries with an ISS of 1–8 (49.1%). The mean ISS for BIs was 12 (SD = 8.9) compared to 5.8 (SD = 1.7) for NBIs (Table 1). Extremities were the body part most prone to BIs (32.5%), followed by the head and face (24.8%; Table 2). There was no significant difference in body region distribution between BIs and NBIs. The head received a higher proportion of severe injuries (AIS ≥ 4) than other body regions (Fig. 2).

**Table 2:** Injuries’ Counts among French Service Members Treated in US Military Medical Treatment Facilities Stratified by Body Region and Injury Classification (Battle vs. Nonbattle)

Body Region	Battle Injuries *n =* 217 (88%)	Nonbattle Injuries *n =* 29 (12%)	Total Injuries *n =* 246	*p-*value
Head	30 (13.8%)	6 (20.7%)	36 (14.6%)	0.34
Face	21 (9.7%)	4 (13.8%)	25 (10.2%)	0.53
Neck	1 (0.5%)	–	1 (0.4%)	1.00
Thorax	30 (13.8%)	2 (6.9%)	32 (13.0%)	0.39
Abdomen	26 (12.0%)	3 (10.3%)	29 (11.8%)	1.00
Spine	6 (2.7%)	3 (10.3%)	9 (3.7%)	0.08
Extremities	73 (33.6%)	7 (24.2%)	80 (32.5%)	0.21
Upper Extremities	32 (14.7%)	4 (13.8%)	36 (14.6%)	0.78
Lower Extremities	41 (18.9%)	3 (10.3%)	44 (17.9%)	0.31
External or Burn	29 (13.4%)	4 (13.8%)	33 (13.4%)	1.00
Unknown	1 (0.5%)	–	1 (0.4%)	1.00

There were a total of 74 admissions to US MTFs along the continuum of care: 12 SMs to Role 2, 54 to Role 3, and 8 to Landstuhl Regional Medical Center Role 4 in Germany. The mean prehospital transportation time was 223.9 min (SD: 284.6): 153.9 min in Afghanistan (SD: 321.9) and 328.8 min during Operation Inherent Resolve (SD: 185.7). However, injury time and arrival time were only available in 42.4% of the records (25 patients). The most frequent transportation method to the 1st facility was by helicopter, used in 61% of patients, followed by ground transport in 7%, while 32% were unknown. Only 9 patients had records of tourniquets (15.3%), with a total of 21 tourniquets placed. At the first admission of the 59 patients, 8.6% were hypothermic (≤ 96.8 °F or 36 °C) out of 43 available records, 5.6% had a systolic blood pressure < 90 mmHg out of 54 available, 9.8% had an oxygen saturation < 90% out of 51 available, 11.8% had a Glasgow Coma Scale < 13 out of 51 available, and 16.3% had a pulse rate higher than 120 out of 53 available records.

**Fig. 3:**
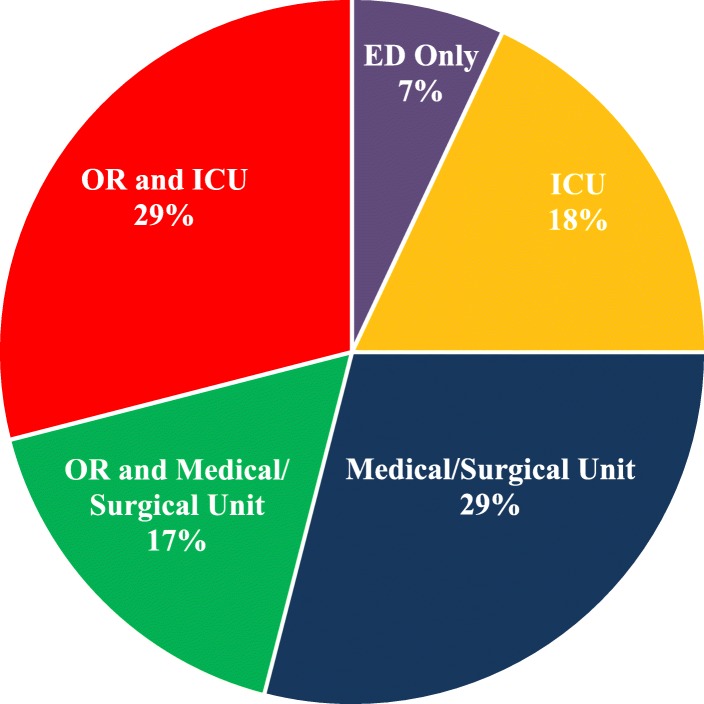
Post Emergency Department Destination of French Service Members Treated in US Military Treatment Facilities. *ED* Emergency Department, *OR* Operation Room, *ICU* Intensive Care Unit, *ED* Only: Returned to Duty, Died, or Transferred to Another Facility

There were 29 diagnostic ultrasonography exams of the abdomen recorded in the registry and 113 computed tomography tests (CT scans), including 32 head, 25 chest, 26 abdominopelvic, and 30 unspecified CT scans. Table 3 provides a detailed summary of the procedures reported per specialty and MTF Role. A total of 173 surgical procedures were recorded in the DoDTR, most of which were performed in Role 3 MTFs (*n =* 143; 83.0%). The most common surgical procedures involved soft tissue and included debridement, suturing, and dressing (39.9%), followed by orthopedic (19.7%), and abdomen procedures (16.7%). Resuscitative fluids were given to 26 French SMs (44.1%), with an average of 4.1 l of fluid per patient; 22 of these patients had BIs. Fourteen patients required blood products (23.7%); 12 of them sustained injuries in battle settings, and those patients received, on average, 25 units of blood. There was no significant difference in the fluids or blood products given to patients with BIs and NBIs (Table 4). In regard to the destinations of the French SMs after the Emergency Department, 46% were admitted to operating rooms (ORs), 47% to Intensive Care Units (ICUs), and 29% were admitted to both the OR and ICU (Fig. 3). The overall length of stay for most French SMs in US MTFs was short: less than 24 h in Role 2 and around one day in Role 3 and Role 4. In regard to the final discharge status reported in the DoDTR, approximately 60% of patients were transferred to other MTFs, either French (*n =* 22; 37.3%), American (*n =* 9; 15.2%), or local MTFs (*n =* 4; 6.8%). There were 19 SMs (32.2%) who returned to duty, one discharged to home (1.7%), and 4 SMs died of their wounds (6.8%): 3 in Role 3 MTFs and one in a Role 4 MTF (Table 5).

**Table 3:** Counts of Surgical Procedures Performed on French Service Members Treated in US Military Medical Treatment Facilities per Level of Care

Procedures	Role 2 *n =* 19 (11%)	Role 3 *n =* 143 (83%)	Role 4 *n =* 11 (6%)	Total *n =* 173
Head (Neurosurgery)	–	4 (2.8%)	–	4 (2.3%)
Face	–	10 (7.0%)	–	10 (5.8%)
Eye	–	9 (6.3%)	–	9 (5.2%)
Dental Procedure	–	1 (0.7%)	–	1 (0.6%)
Thorax	–	15 (10.5%)	–	15 (8.7%)
Chest Tube	–	7 (4.9%)	–	7 (4.0%)
Emergency Resuscitative Thoracotomy	–	5 (3.5%)	–	5 (2.9%)
Explorative Thoracotomy	–	1 (0.7%)	–	1 (0.6%)
Lung Resection	–	1 (0.7%)	–	1 (0.6%)
Diaphragm Suture	–	1 (0.7%)	–	1 (0.6%)
Abdomen	2 (10.5%)	22 (15.4%)	5 (45.5%)	29 (16.7%)
Laparotomy	1 (5.3%)	6 (4.2%)	2 (18.2%)	9 (5.2%)
Laparoscopy	–	2 (1.4%)	–	2 (1.1%)
Bowel	1 (5.3%)	6 (4.2%)	1 (9.1%)	8 (4.6%)
Liver	–	1 (0.7%)	–	1 (0.6%)
Splenectomy	–	1 (0.7%)	–	1 (0.6%)
Urological Surgery	–	4 (2.8%)	–	4 (2.3%)
Other	–	2 (1.4%)	2 (18.2%)	4 (2.3%)
Orthopedics	9 (47.4%)	23 (16.1%)	2 (18.2%)	34 (19.7%)
Amputation	1 (5.3%)	5 (3.5%)	–	6 (3.5%)
Arthroscopy	–	1 (0.7%)	–	1 (0.6%)
External Fixation	2 (10.5%)	6 (4.2%)	–	8 (4.6%)
Fasciotomy	–	6 (4.2%)	1 (9.1%)	7 (4.1%)
Hand Surgery	1 (5.3%)	3 (2.1%)	–	4 (2.3%)
Other	5 (26.3%)	2 (1.4%)	1 (9.1%)	8 (4.6%)
Vascular	1 (5.3%)	11 (7.7%)	–	12 (2.9%)
Repair	–	5 (3.5%)	–	5 (2.9%)
Ligation	1 (5.3%)	4 (2.8%)	–	5 (2.9%)
Shunt or Bypass	–	2 (1.4%)	–	2 (1.1%)
Soft tissue (Debridement, Suture, Dressing)	7 (36.8%)	58 (40.5%)	4 (36.3%)	69 (39.9%)

**Table 4:** Counts and Percentages of French Service Members Receiving IV Fluids and Blood Products per Battle Status. Counts and Ranges (Lowest and Highest) of IV Fluids (in Liters) and Blood Products (in Units) are Provided

Blood Products and Solutions	Battle *n =* 46 (78%)	Nonbattle *n =* 13 (22%)	Total *n =* 59	*p*-value
IV Fluids				
Crystalloid recipients	22 (47.8%)	4 (30.8%)	26 (44.1%)	0.35
Crystalloid units	99.4 (0.35–13.6)	4.2 (0.5–1.9)	103.6 (0.35–13.6)	
Colloid recipients	4 (8.7%)	–	4 (6.8%)	0.56
Colloid units	3 (0.25–1.75)	–	3 (0.25–1.8)	
Total recipients	22 (47.8%)	4 (30.8%)	26 (44.1%)	0.35
Total units	102.4 (0.35–15.3)	4.2 (0.5–1.9)	106.6 (0.35–15.3)	
Blood Products				
Whole Blood recipients	1 (2.2%)	–	1 (1.7%)	1.00
Whole Blood units	4	–	4	
PRBC^a^ recipients	11 (23.9%)	1 (7.7%)	12 (20.3%)	0.27
PRBC units	121 (4–30)	7	128 (4–30)	
Platelets recipients	6 (13%)	–	6 (10.2%)	0.32
Platelets units	17 (1–6)	–	17 (1–6)	
Cryoprecipitate recipients	5 (10.9%)	–	5 (8.5%)	0.58
Cryoprecipitate units	86 (1–40)	–	86 (1–40)	
FFP^b^ recipients	12 (26.1%)	1 (7.7%)	13 (22%)	0.26
FFP units	111 (3–23)	4	115 (3–23)	
Total recipients	13 (28.3%)	1 (7.7%)	14 (23.7%)	0.16
Total units	339 (4–73)	11	350 (4–73)	

*PRBC* Packed Red Blood Cell, *FFP* Fresh Frozen Plasma

**Table 5:** Discharge Status of French Service Members Treated in US Military Treatment Facilities per Discharging Level of Care

Discharge Status	From Role 2 *n =* 4 (6.8%)	From Role 3 *n =* 46 (78.0%)	From Role 4 *n =* 9 (15.25%)	Total *n =* 59
Transferred to US MTF	–	9 (19.6%)	–	9 (15.2%)
Transferred to French MTF	3 (75.0%)	15 (32.6%)	4 (44.4%)	22 (37.3%)
Transferred to Local MTF	–	4 (8.7%)	–	4 (6.8%)
Returned to Duty	1 (25.0%)	14 (30.4%)	4 (44.4%)	19 (32.2%)
Discharged to Home	–	1 (2.2%)	–	1 (1.7%)
Death^a^	–	3 (6.5%)	1 (11.2%)	4 (6.8%)

^a^.Death after Admission to US Military Treatment Facilities

## Discussion

Casualties of the Global War on Terror continue to occur in many areas in the world where the NATO coalition deploys armed forces. France is one of the main contributors to NATO in these conflicts and has suffered its share of casualties. In this study, we examined the 59 French SMs who were, at one point, treated in Roles 2, 3, or 4 US MTFs. The exclusive distribution of French casualties in the DoDTR in Afghanistan until 2014 and in the Middle East (Iraq and Syria) beginning in 2015 corresponds to the periods when France took part in the conflicts in these countries. The French casualties recorded in the DoDTR in Afghanistan reached a peak in 2009, with 15 battle-related casualties treated in US MTFs that year. However, starting in 2010, we observed a decrease of French casualties in the DoDTR, despite a reported increase in fatalities by the French Joint Staff (*Etat-Major des* Armées) [16] over the same period in Afghanistan. This antithetical decline may be attributable to the opening of a French Role 3 MTF in Kabul International Airport (KaIA), which took place in July 2009 in the capital region of Afghanistan; this is where the main body of the French troops was deployed at that time [3, 4]. Therefore, the majority of French casualties were treated in this French facility. However, the decline in the number of French SMs treated in US MTFs could be due to other reasons, such as changes in operational tempo.

BIs tend to be more severe and predominantly penetrating in nature, and the main mechanism of injury was explosions. The patients in this group were on average 6 years younger compared to their counterparts with NBIs, probably due to the fact that SMs directly involved in the fights are usually younger, whereas those with higher ranks and in command positions are usually older. Meanwhile, NBIs tend to be blunt in nature, with motor vehicle crash being the most common mechanism of injury. Extremities were the most affected body region, especially in BIs where these injuries account for half of the casualties. However, head injuries were the most severe, followed by thorax injuries. The aforementioned demographic findings are consistent with previous studies [1, 13, 17–19].

Prehospital transportation time was found to be relatively long, exceeding the timeframe for the “golden hour”; however, these data were poorly recorded (only 42.4% were available). The NATO doctrine of medical support to operations recommends a timeline of one hour for damage control resuscitation and one to two hours for damage control surgery [20, 21]. However, decreasing transportation time in accordance with the “golden hour” principle has been shown to be significantly associated with reduced mortality among US casualties since the US mandate in 2009 [22]. BIs required more surgical procedures, intravenous fluids (IV), and blood products. Most of the procedures performed were in Role 3 US MTFs, given their advanced surgical capabilities compared to Role 2 MTFs [23, 24]. Similar to the findings in other studies, a large proportion of casualties went directly from point of injury to Role 3 [9]. Orthopedic and soft tissue represented 60% of all procedures, consistent with previous findings [25]. This emphasizes the need for orthopedic surgeons in deployed MTFs [18]. Nearly all intravenous fluids and blood products were given to patients with BIs. It is worth mentioning that US MTFs do not use freeze-dried plasma, which has been a staple of French combat casualty care [26].

We encountered a number of limitations in this study. Primarily, this study is retrospective, and the patient population studied was small and may not be representative of all French SMs injured in Afghanistan and the Middle East. The SMs in the study may have different characteristics than those treated in French MTFs, or those who were treated in other NATO MTFs. No data has been collected on the reasons why these SMs were brought to US MTFs to be treated, nor is there data available regarding the geographic locations of the injuries, especially in the time period between 2009 and 2013 when KaIA was an operating French MTF. The DoDTR is not designed to collect such information, making it difficult to make inferences based on physical proximity in regard to their admissions to US MTFs. However, “mass casualty” events may require evacuating wounded soldiers to several MTFs. For example, in January 2012, both French and US Role 3 MTFs received casualties from a massive shooting attack on a French operational base [27]. Therefore, we can expect that the geographic vicinity of the US MTFs to the point of injury, along with bed availability and other logistic reasons are possible explanations. Like other registries, the DoDTR also suffers from missing data. The data are abstracted from medical records and depend on the accuracy of the recorded information by deployed healthcare professionals. Prehospital data are the most difficult to document and capture [28].

Nevertheless, this study provides a descriptive summary of a population not previously studied. Although not necessarily representative of all injured French soldiers during the same period, the pattern of injuries in this population is valuable information. There are many studies that have been conducted concerning US SMs treated in US MTFs and French SMs treated in French MTFs, but, to our knowledge, there are no studies that aim to examine French SMs treated in US MTFs. This missing information in the care provided to French SMs can, in the future, be combined with their French medical records (if available) in order to provide a complete picture of their injuries, the care provided to them, and their overall outcomes and complications. This study reflects the multinational nature of the medical support now being part of modern operations, where the responsibility of military medical support is increasingly shared by allied nations [29]. Data collection in military operations continues to be a challenge, and the DoDTR represents the largest military trauma data source of its kind. France is currently working on creating a similar military trauma registry in order to collect accurate data on its injured soldiers at deployed French MTFs in order to support on-going performance improvement and injury prevention efforts in combat casualty care. The DoDTR, with its extensive data on traumatic injuries and care provided, offers a compelling model from which the French Military can benefit in building a trauma registry to provide the best possible care to French SMs.

## Conclusions

The majority of injuries sustained by French SMs derived from of battle-related explosive causes and exhibited a predominance of extremities injuries patterns similar to those in combat zones reported by other NATO nations. The DoDTR provides extensive data on traumatic injuries and the care provided in US MTFs, which can be used to inform injury prevention and to improve combat casualty care. This emphasizes the need to establish a French Military trauma registry similar to the DoDTR to record traumatic injuries sustained by French SMs and that is collected by deployed French MTFs to allow performance improvement measures and improve combat casualty care.

## Abbreviations

BI: Battle Injury
DoDTR: Department of Defense Trauma Registry
IV: Intravenous
JTS: Joint Trauma System
MTF: Military Treatment Facility
NATO: North Atlantic Treaty Organization
NBI: Nonbattle Injury
SM: Service Member
US: United States
