# Correction to: Description of trauma among French service members in the Department of Defense Trauma Registry: understanding the nature of trauma and the care provided

**DOI:** 10.1186/s40779-019-0211-z

**Published:** 2019-07-28

**Authors:** Marc A. Schweizer, Jud C. Janak, Zsolt T. Stockinger, Tristan Monchal

**Affiliations:** 1 United States Department of Defense Joint Trauma System, Joint Base San Antonio Fort Sam Houston, Houston, TX 78234 USA; 2 Naval Medical Readiness Training Command Jacksonville, Jacksonville, FL 32212 USA; 3 Sainte Anne Military Hospital, BP 600, 83800 Toulon, Cedex 9 France

**Correction to: Mil Med Res**

**https://doi.org/10.1186/s40779-019-0197-6**

After publication of this article [1], it was brought to our attention that the Fig. 2 is incorrect. The correct Fig. 2 is as below:

**Fig. 2:**
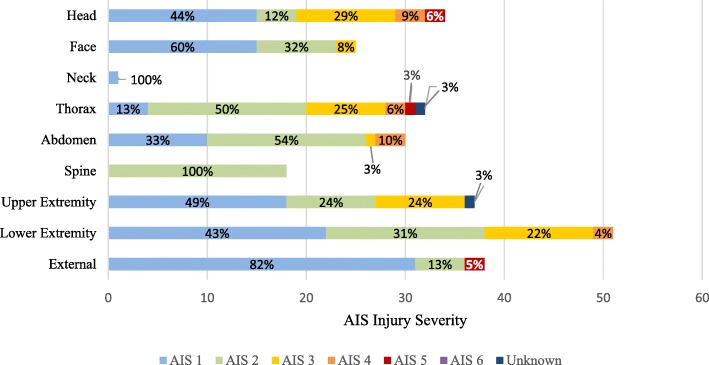
Proportions of injuries among French Service Members treated in U.S. Medical Treatment Facilities per Body Region and AIS severity. *AIS* Abbreviated Injury Scale
